# Systemic delivery of HER2-retargeted oncolytic-HSV by mesenchymal stromal cells protects from lung and brain metastases

**DOI:** 10.18632/oncotarget.5793

**Published:** 2015-09-27

**Authors:** Valerio Leoni, Valentina Gatta, Arianna Palladini, Giordano Nicoletti, Dario Ranieri, Massimiliano Dall'Ora, Valentina Grosso, Martina Rossi, Francesco Alviano, Laura Bonsi, Patrizia Nanni, Pier-Luigi Lollini, Gabriella Campadelli-Fiume

**Affiliations:** ^1^ Department of Experimental, Diagnostic and Specialty Medicine, University of Bologna, Bologna, Italy

**Keywords:** oncolytic herpes simplex virus, viral retargeting, mesenchymal stem cells, systemic delivery, metastases

## Abstract

Fully retargeted oncolytic herpes simplex viruses (o-HSVs) gain cancer-specificity from redirection of tropism to cancer-specific receptors, and are non-attenuated. To overcome the hurdles of systemic delivery, and enable oncolytic viruses (o-viruses) to reach metastatic sites, carrier cells are being exploited. Mesenchymal stromal cells (MSCs) were never tested as carriers of retargeted o-viruses, given their scarse-null expression of the cancer-specific receptors. We report that MSCs from different sources can be forcedly infected with a HER2-retargeted oncolytic HSV. Progeny virus spread from MSCs to cancer cells *in vitro* and *in vivo*. We evaluated the organ distribution and therapeutic efficacy in two murine models of metastatic cancers, following a single i.v. injection of infected MSCs. As expected, the highest concentration of carrier-cells and of viral genomes was in the lungs. Viral genomes persisted throughout the body for at least two days. The growth of ovarian cancer lung metastases in nude mice was strongly inhibited, and the majority of treated mice appeared metastasis-free. The treatment significantly inhibited also breast cancer metastases to the brain in NSG mice, and reduced by more than one-half the metastatic burden in the brain.

## INTRODUCTION

Viruses belonging to different families have been chosen, and, in many cases, genetically modified to generate replicating oncolytic agents that target tumor cells with varying extent of cancer-specificity [1, 2]. A number of oncolytic viruses (o-viruses) which induced tumor regression in animal models of human cancers are now being assayed in clinical trials [3]. Therapeutic effects were reported in recent years against a number of tumors, including glioblastoma, B cell malignancy, metastatic melanoma, liver cancer [4-8]. The major obstacles that are being faced with the translation of oncolytic viruses to the clinics are the routes of delivery, and the immune response that pre-exists, and/or is induced by the viral vector, and limits the efficacy of the treatments as well as repeated administrations [2].

A number of features make HSV a highly promising o-virus, in particular the large genome space which enables the insertion of heterologous sequences, opportunity of genetic manipulations, possibility to control any unwanted replication in humans in a worst-case scenario, etc [1, 2, 9]. On the other hand, o-HSVs may exhibit a non optimal ability to systemic dissemination. The first generation o-HSVs, now in clinical trials, are conditional replication deletion or natural mutants. Their replication occurs preferentially in subsets of cancer cells which are defective in specific branches of the innate response; their replication in non-cancer cells is counteracted by the host defence. Their drawbacks are the attenuation introduced in order to confer cancer-specificity and for safety reasons, and their non-stringent cancer-specificity. To overcome these limitations, second generation o-HSVs encode the cytokines GM-CSF, or IL12, in order to stimulate the antitumor activity of the immune system of host [10-13]. The prototype is T-VEC, formerly named Oncovex(GM-CSF), an attenuated virus [10]. In a phase 3 clinical trial, T-VEC led to an improved outcome in patients carrying metastatic melanoma and treated intratumorally [4].

An alternative approach to attenuation has been the design of o-HSVs fully retargeted to cancer-specific receptors and detargeted from natural receptors [9, 14-18]. The high degree of cancer-specificity attained by this approach makes it unnecessary to introduce deletions or mutations in virulence genes. These viruses preserve the full lytic activity of wt-HSVs. Our laboratory has generated o-HSVs fully retargeted to the HER-2 cancer receptor, by engineering single-chain antibody (scFv) to HER-2 in gD. The engineering entails deletions of appropriate regions in gD responsible for binding the natural gD receptors, nectin1 and HVEM, and their replacement with scFv. The R-LM249 and R-LM113 recombinants carry the scFv to HER-2 inserted in place of the 60-218, or 6-38 AA regions in gD, respectively [16, 17]. When administered intratumorally, both viruses exerted antitumor activity in athymic nude mice bearing HER2+ breast or ovary cancers, or in immunocompetent mice model of HER2+ glioblastoma [19-22]. R-LM249 was administered also intraperitoneally and exerted a therapeutic effect against the peritoneal carcinomatosis from ovary cancer, and against ovary and brain metastases from human breast cancer [22].

Ideally, in humans, the o-virus should be delivered systemically, so that it can reach distal metastases. In the case of o-HSVs, the efficacy of the intravenous (i.v.) systemic route of administration has been attempted in a limited number of preclinical studies [11, 23-25]. In humans, o-HSV was safely administered through hepatic artery to patients with hepatic colorectal metastases [26-28]. Yet, the i.v. systemic administration may turn out not to be practicable for a number of reasons. It may be very difficult, and most likely unsafe, to reach the blood concentrations required to achieve the locally active concentrations, given that the majority of systemically administered o-viruses are rapidly cleared by parenchymal organs and inactivated by non-specific and specific defence systems. Furthermore, there would be production problems [29]. A recent approach to circumvent these obstacles envisions the use of carrier cells, which package the viral cargo and deliver it to the tumor. Initial studies employed irradiated tumor cells, which have not been pursued because of safety concerns [30-33]. The o-viruses can be packaged by carrier cells in two different ways. It can be loaded onto the cells, or taken into cells compartments; because it does not undergo replication, the o-virus is delivered in limited amounts. Alternatively, the carrier cell sustains o-virus replication prior to its delivery to the tumor, and thus amplifies the payload. In turn, carrier cells deliver their cargo in three ways [32]. In some cases, tumor-specific carrier T-cells or cytokine-induced killer carrier cells target tumor cells specifically. Alternatively, carrier cells do not target the tumor cells, but the tumor location, e.g. lymphnodes. As a third possibility, carrier cells may be attracted to the tumor microenvironment. The promising mesenchymal stem cells belong to the latter group; they accumulate within the tumor stroma because of hypoxic conditions and of tumor-associated expression of inflammatory chemokines. Major limits are that the cancer-specificity of the o-viruses largely prevents infection of these cells. Secondly, the adoptively transferred mesenchymal stem cells may contribute to feed the tumor. So far, mesenchymal stem cells have been assayed as carrier of Adenovirus, measles virus and reolysin. Only in very few studies they were evaluated as carrier of HSV, for intraperitoneal delivery, or for local delivery to brain tumors [32, 34-40].

The delivery of fully detargeted/retargeted o-viruses by mesenchymal stromal cells (MSCs) has not been attempted with any virus, nor has been the i.v. systemic delivery of o-HSVs by carrier cells. The aim of this work was to provide proof-of-principle that the i.v. systemic delivery of R-LM249 via MSCs is feasible and effective. An obstacle was that the HER2-retargeted R-LM249 can hardly infect MSCs, which exhibit low-null expression of the HER2 oncogene. We report that MSCs can be forcedly infected with the retargeted R-LM249 and that a single i.v. administration of R-LM249-infected MSCs exerted therapeutic effects against models of lung and brain metastases.

## RESULTS

### Infection with the HER2-retargeted R-LM249 is greatly enhanced upon exposure of virus-cell mixture to the fusogenic agent PEG6000

MSCs cells were derived from human placenta, dental pulp, adipose tissue, bone marrow, as described [41-43]. The ones derived from placenta (fetal membrane) (FM-MSCs) were characterized as MSCs, based on expression of the stromal markers CD90, CD44, CD105, CD73, lack of expression of the CD34 and CD45 hematopoietic markers, as well as of HLA class II (Figure S1 A), and ability to undergo osteogenic, adipogenic, or chondrogenic differentiation upon appropriate treatments (Figure S1 B-E).

**Figure 1:**
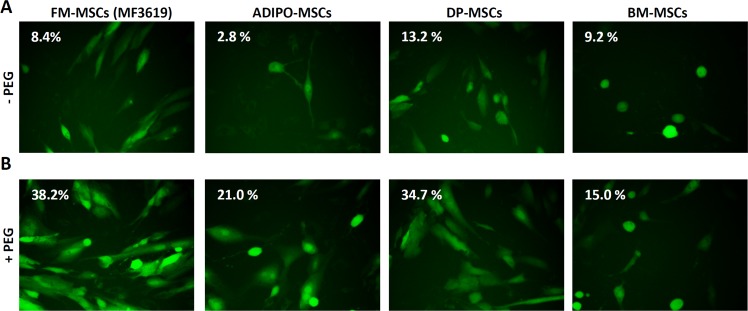
Enhanced infection of FM-MSCs with R-LM249 by means of PEG6000 **A.**, **B.** Enhanced infection of FM-MSCs with R-LM249 by aid of PEG6000. Virions were absorbed to FM-MSCs at 10 PFU/cell. The virion-cell mixture was exposed for 20 sec to PEG6000 (panel B) or left without PEG6000 treatment (panel A). Infection was monitored through detection of EGFP engineered in the viral genome by fluorescence microscopy or by flow cytometry. Figures within micrographic images indicate the percentage of infected cells, as determined by flow cytometry.

HER2 expression was rather low in all analyzed MSCs as compared to HER2-positive SK-OV-3 ovary cancer cells (Figure S2 A). In agreement with the low HER2 expression, FM-MSCs were infected at very low efficiency with the HER2-retargeted HSV, named R-LM249, at 1 or 10 PFU/cell (Figure 1A and Figure S2 B, and Table 1), as detected through the enhanced green fluorescent protein (EGFP) marker inserted in the viral genome (Figure 1A and Figure S2 B), or flow cytometry (Table 1). To facilitate infection, R-LM249 was allowed to absorb to cells, and virion-to-cell fusion was promoted by exposure of the cells, carrying the absorbed virus, to the fusogenic agent PEG6000. For comparison, we made use of J cells, which fail to express any receptor for HSV gD, and are therefore resistant to infection [44]. Figures 1B and S2 C show that a 20 sec exposure to 40% PEG6000 greatly facilitated infection of both FM-MSCs and J cells. Also the MSCs from adipose tissue, a source of MSCs suitable to autologous use in patients, could be infected with R-LM249 *via* PEG6000 (Figure 1A-1B). The MSCs from additional sources - dental pulp, bone marrow (Figure 1), an independent batch of FM-MSCs named MF3620 (Figure S2) - exhibited varying, but consistently low levels of infection, and were efficiently infected when exposed to PEG6000 (Table 1, Figure 1 and Figure S2). We conclude that exposure to PEG6000 of MSCs from different origins is a generally suitable treatment to greatly facilitate infection with the HER2-retargeted R-LM249. This legitimates the use of the FM-MSCs, which were available in higher quantity.

**Table 1:** R-LM249 infection of MSCs derived from various tissues is enhanced by PEG6000

	Percentage of infected cells^a^			
	1 PFU/cell^b^		10 PFU/cell^b^	
**PEG treatment**	**-^c^**	**+^c^**	**-^c^**	**+^c^**
**Cell line**				
**FM-MSCs (MF3619)**	3.2	26.5*	8.4	38.2^
**FM-MSCs (MF3620)**	0.9	11.0*	3.6	23.8^
**ADIPO-MSCs**	0.8	12.0*	2.8	21.0^
**Dental pulp-MSCs**	6.4	22.2*	13.2	34.7^
**Bone marrow-MSCs**	1.7	5.9*	9.2	15.0^
**SK-OV-3**	90.9	85.8	93.1	92.4
**J**	0.1	3.1	0.3	13.8

a Extent of R-LM249 infection determined by flow cytometry

b Input MOI

c ± PEG treatment

*,^ p<0.01, one-tailed paired *t* test between untreated and PEG-treated MSCs of all origins.

### R-LM249 replicates in MSCs and progeny virus spreads to the HER2+ cancer cells *in vitro* and *in vivo*

**Figure 2:**
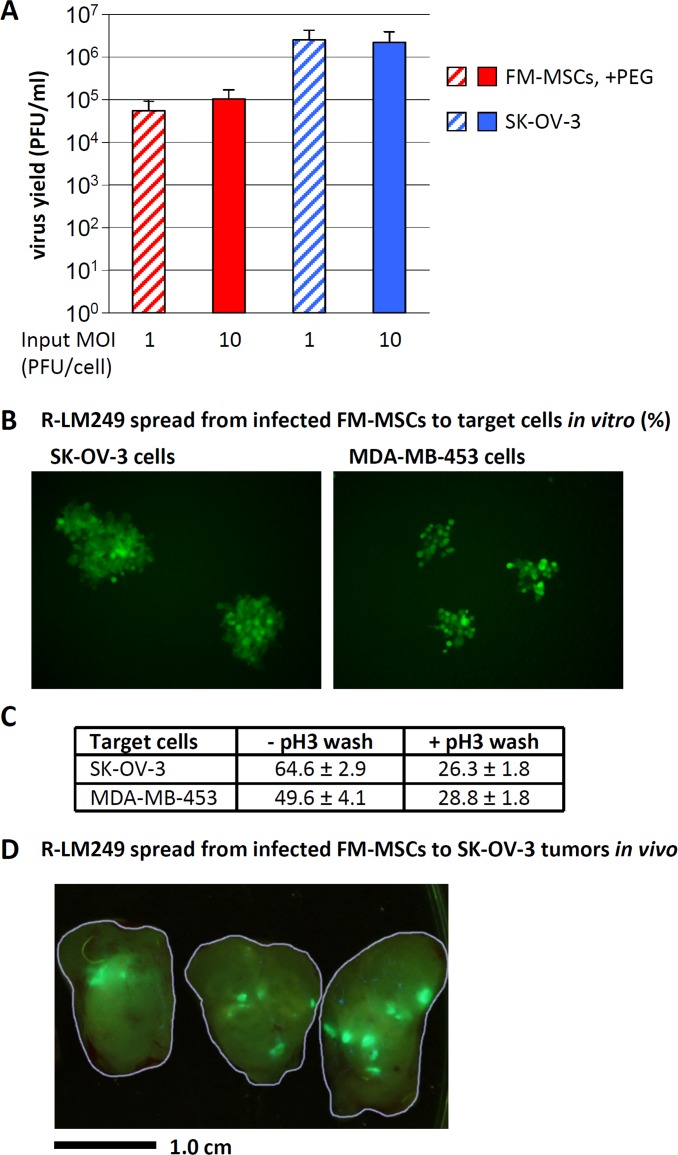
R-LM249 replicates in FM-MSCs, and progeny virus spreads to SK-OV-3 or MDA-MB-453 cancer cells *in vitro* and *in vivo* **A.** Yield of R-LM249 in FM-MSCs cells. FM-MSCs and SK-OV-3 cells were infected with R-LM249 at input MOI of 1 or 10 PFU/cell. Infection of FM-MSCs was enhanced by PEG6000. Progeny virus was harvested at 24 h after infection, and titrated in SK-OV-3 cells. Figures represent the average of triplicate samples, ± S.D. **B.**, **C.** Spread of progeny R-LM249 from FM-MSCs to HER2-positive cancer cells. FM-MSCs cells, infected as in panel A, rinsed or not with a pH 3 buffer at 3 h after PEG6000 treatment, were trypsinized at 7 h after infection, and plated onto monolayers of SK-OV-3 cells or MDA-MB-453 cells. Spread of infection was quantified as number of plaques, detected by fluorescence microscopy 48 h later. **C.** The number of plaques was expressed as percentage of the seeded infected cells. Figures represent the average of triplicate samples, ± S.D. **D.** Intratumor spread of EGFP-encoding virus 6 days after the injection of 10^6^ infected FM-MSCs into a SK-OV-3 s.c. tumor. The contours of three slices of a dissected tumor are outlined in grey. Bar = 1 cm.

We verified whether R-LM249 replicates in FM-MSCs and releases infectious progeny. FM-MSCs were infected with R-LM249, at 1 or 10 PFU/cell *via* PEG6000. Progeny virus was harvested at 24 h after infection and titrated in SK-OV-3 cells. For comparison, we measured R-LM249 replication in SK-OV-3 cells, the cells usually employed to produce virus stocks. The 24 h yield appeared to be 1.5 - 2 Log lower in FM-MSCs than in SK-OV-3 cells (Figure 2A). By taking into account that the SK-OV-3 cultures contained about 6-fold more cells than the FM-MSCs cultures, and that, in the latter cultures, only a fraction of cells (30-40%) was infected, the estimated yield/cell in FM-MSCs was in the same order of magnitude as that in SK-OV-3 cells.

R-LM249 produced in FM-MSCs was released and spread to monolayers of HER2 positive SK-OV-3 or MDA-MB-453 cancer cells. This property was verified by means of an infectious centre assay. FM-MSCs infected with R-LM249 *via* PEG6000 were trypsinized, exposed or not to pH 3 rinse to remove any absorbed virus, and seeded onto a monolayer of target SK-OV-3 or MDA-MB-453 cells. The plaques were scored at 48 h (Figure 2B). The efficiency of spread was expressed as the percentage number of plaques relative to the number of seeded infected cells (Figure 2C). Two features are worth noting. The efficiency of spread of progeny virus was about 25%; it is likely that the manipulations inactivated a fraction of the infected cells. Secondly, some virus remained absorbed to cell surfaces, and was inactivated by the pH 3 wash. Next, we verified whether R-LM249 could spread from infected FM-MSCs to xeno-transplanted tumors *in vivo*. SK-OV-3 cells were subcutaneously transplanted in athymic nude mice. Five weeks later, FM-MSCs were infected with R-LM249 *via* PEG6000 *ex vivo*. Seven h later, cells were trypsinized and injected intratumorally. Tumors were examined 6 days after the injection of infected MSCs. Figure 2D shows that the virus released from MSCs indeed colonized the tumor and replicated.

Finally, we evaluated the extent of cell surface expression of R-LM249 chimeric gD at 8, 12, 24 h after infection. Infected FM-MSCs exhibited a weak fluorescence relative to infected SK-OV-3 cells (Figure S3).

Cumulatively, these data indicate PEG6000-enhanced infection of MSCs with R-LM249 led to a productive infection, and the progeny virus could spread to cancer cells *in vitro* and *in vivo*. All subsequent *in vivo* experiments were performed with FM-MSCs infected with R-LM249 by way of PEG6000.

### Tissue distribution of R-LM249 delivered *via* carrier MSCs in athymic nude mice

**Figure 3:**
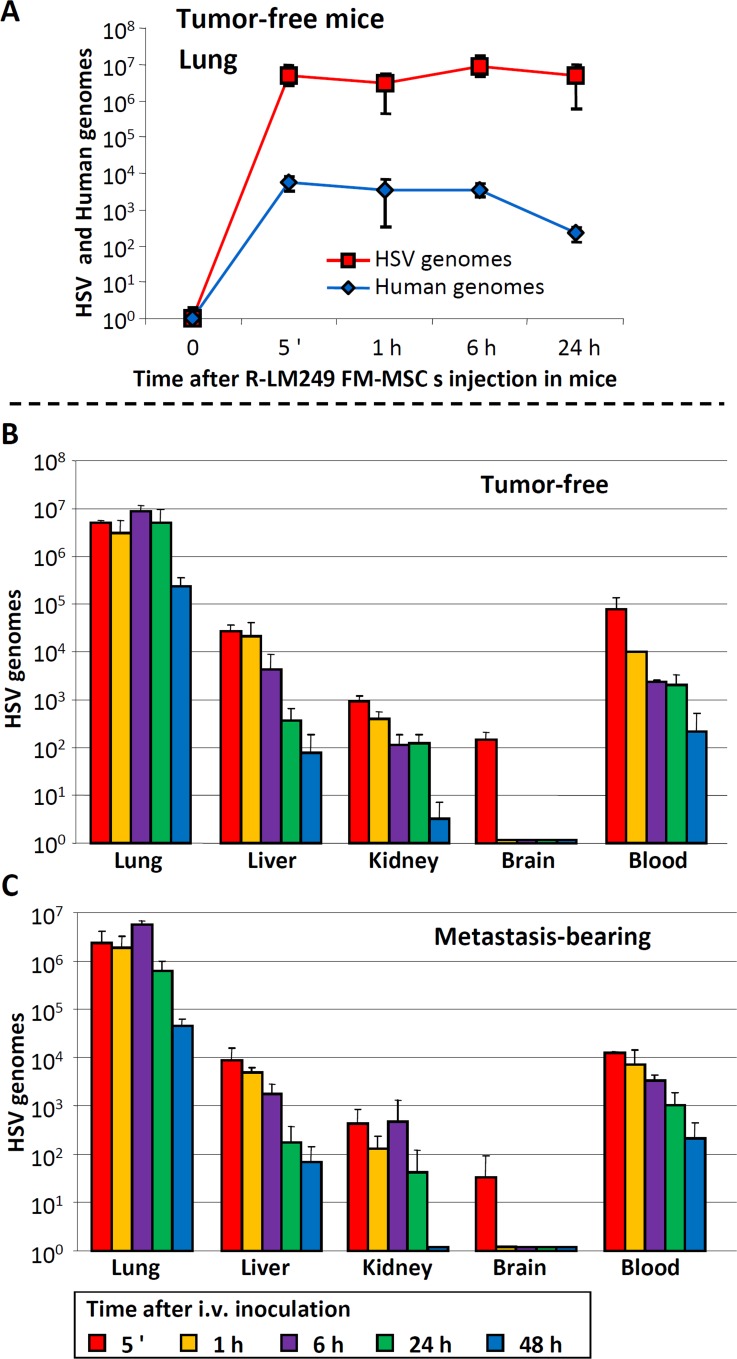
Distribution of R-LM249-infected FM-MSCs to lungs, blood and other organs of tumor-free and metastasis-bearing athymic nude mice Athymic nude mice were inoculated i.v. with SK-OV-3 cells, or left untreated. Nine weeks later, FM-MSCs were infected *ex vivo* with R-LM249 by aid of PEG6000. Seven h later they were administered i.v. to metastasis-bearing or tumor-free mice. Lungs, blood and other organs were withdrawn at the indicated times after infected cell administration. DNA was extracted. Samples were analyzed by quantitative PCR, with primers annealing to the HSV genome (HSV) [22], or human genome (human). **A.** Kinetics of HSV and human genome copy numbers in lungs of tumor-free mice, sacrificed at the indicated times after i.v. administration of R-LM249-infected FM-MSCs. **B.**, **C.** Kinetics of viral genome copy numbers in the indicated organs of tumor-free **B.** and metastasis-bearing **C.** mice. Results represent the average of 3 mice for each time point ± S.D.

To investigate the distribution of R-LM249-infected FM-MSCs injected i.v., and the ensuing delivery of R-LM249, we determined by q-PCR the kinetics of human and viral genome copy numbers in various anatomical sites. In healthy athymic nude mice, i.v. injection of R-LM249-infected FM-MSCs produced the highest concentrations of cellular and viral genomes in the lungs (Figure 3A-3B). Since the presence of metastatic nodules, especially in the lungs, could affect the distribution of both carrier cells and virus, we determined the distribution of viral genomes in athymic nude mice inoculated i.v. with SK-OV-3 carcinoma cells, which produce lung metastases. Six weeks later mice were treated with i.v.-injected R-LM249-infected FM-MSCs. In this case, the evaluation of human genomes provided a cumulative measure of MSCs and of metastatic cells, both of human origin. The kinetics of viral genomes over the first 24 h was similar in tumor-free and metastasis-bearing mice, regardless of the metastatic burden (Figure 3B-3C). These results indicate that the efficiency of R-LM249 delivery to the lungs by infected FM-MSCs was independent of metastatic burden, a feature consistent with the low cell surface expression of chimeric gD in infected FM-MSCs, which prevented a specific interaction of the infected carrier cells with the target tumor cells.

The analysis of viral genomes circulating in the bloodstream of healthy and metastasis-bearing mice also showed overlapping kinetics (Figure 3B-3C). Since free virions are rapidly taken up from the blood, the persistence over the analyzed time interval suggests that infected carrier cells remain in blood stream and can potentially deliver their viral cargo to organs. Liver and kidneys contained several orders of magnitude less viral genomes than the lungs, as expected; brains were almost negative, except in the very first minutes after infection (Figure 3B-3C). The genome copy numbers decreased over 48 h. On the whole, the results of extrapulmonary localizations indicate that the R-LM249 delivered though infected MSCs to the lungs, and present in the bloodstream, could rapidly reach parenchymal organs and persisted systemically for 2 days, potentially targeting metastatic lesions in body districts.

### Therapy of lung metastases by i.v.-administered carrier MSCs infected with R-LM249

To test the therapeutic activity of carrier cell-delivered R-LM249, we generated lung metastases in athymic nude mice by the i.v. injection of SK-OV-3 ovarian carcinoma cells. The model mimics the systemic spread of human ovarian cancer, including the wide heterogeneity in the number of metastatic lung nodules, which in mice ranged from zero to more than 200. Seven days after the injection of tumor cells, mice were treated with a single i.v. administration of R-LM249-infected FM-MSCs. For ethical reasons, mice were sacrificed at a fixed time after tumor cell injection, before metastases could cause distress and pain; therefore, we did not evaluate overall survival. R-LM249 significantly inhibited metastatic development, reducing to zero the median number of lung metastases (Table 2). The therapeutic activity was entirely attributable to the o-HSV, since treatment with uninfected FM-MSCs did not modify either metastasis incidence or their number. These data provide the first evidence that R-LM249 exerts anti-tumor activity against lung metastases of HER-2-positive carcinoma upon a systemic administration *via* carrier MSCs.

**Table 2:** Incidence of lung metastases in athymic nude mice treated with R-LM249-infected FM-MSCs

Treatment^a^	Lung metastases		
	Incidence (%)	Median	Range
**Untreated**	13/19 (68%)	**5**	0 – 157
**FM-MSCs**	13/18 (72%)	**9.5**	0 - >200
**R-LM249 FM-MSCs**	3/19* (16%)	0*	0 - 31

a Mice received an i.v. injection of SK-OV-3 cells; one week later they received an i.v. injection of R-LM249-infected FM-MSCs (R-LM249 FM-MSCs), uninfected FM-MSCs (FM-MSCs), or vehicle (Untreated). Mice were sacrificed 9 weeks later.

* Significance of difference versus Untreated and FM-MSCs is p<0.01 (Fisher's Exact test for frequency and Mann–Whitney rank sum test for metastasis number).

### Therapy of brain metastases of mammary carcinoma

**Figure 4:**
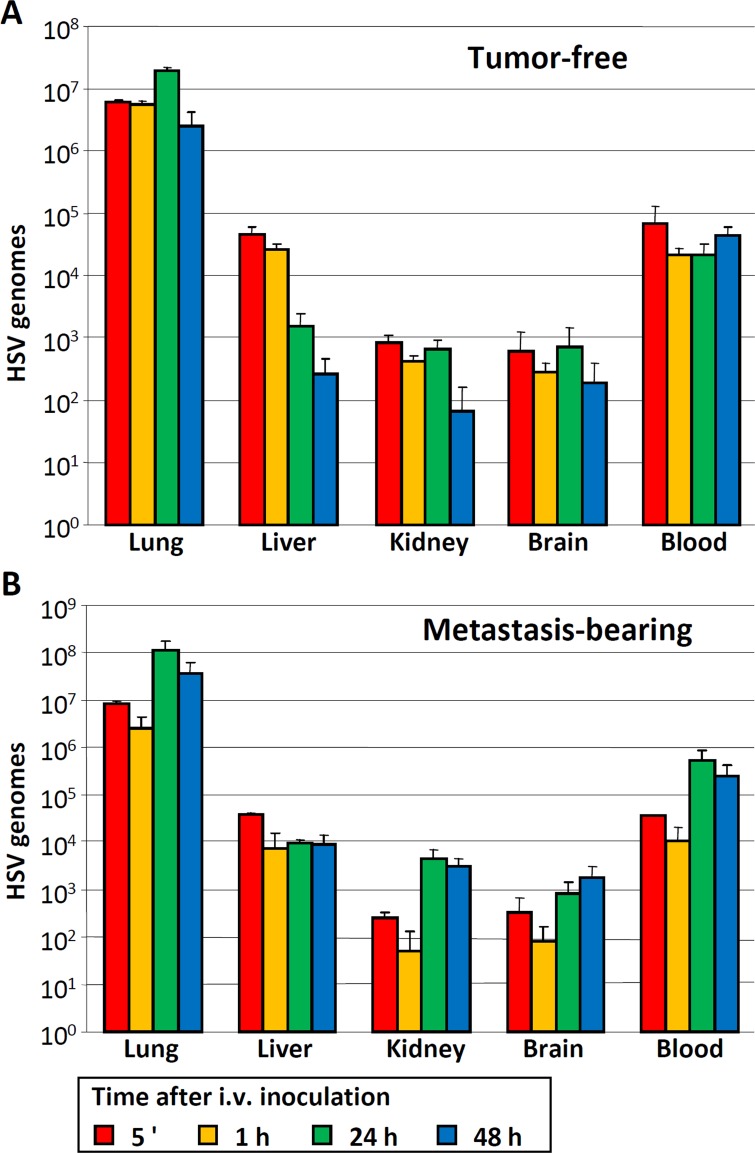
Distribution of HSV genomes to blood and other organs of tumor-free and metastasis-bearing NSG mice **A.**, **B.** NSG mice were inoculated i.v. with MDA-MB-453-EGFP cells, or left untreated. Six weeks later, FM-MSCs were infected *ex vivo* with R-LM249 by aid of PEG6000. Seven h later they were administered i.v. to tumor-free **A.** or metastasis-bearing **B.** mice. The indicated organs were withdrawn at the indicated times after infected cell administration. DNA was extracted and samples were analyzed by qPCR with primers annealing to the HSV genome (HSV) [22]. Results represent the average of 3 mice for each time point ± S.D.

**Figure 5:**
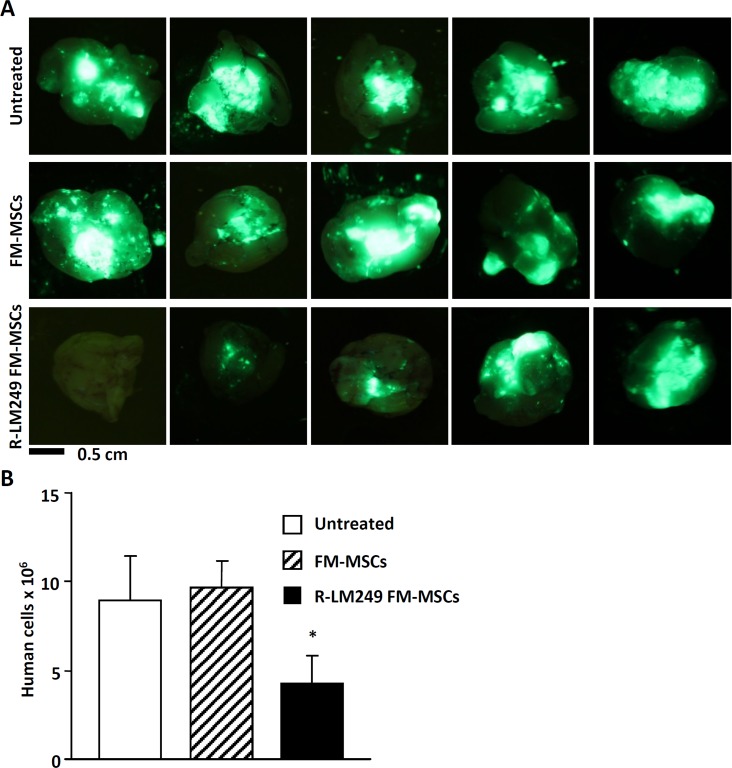
Therapy of brain metastases with R-LM249-infected FM-MSCs Mice received an i.v. injection of MDA-MB-453-EGFP cells. One week later, they received an i.v. injection of R-LM249-infected FM-MSCs (R-LM249 FM-MSCs), uninfected FM-MSCs (FM-MSCs), or vehicle (Untreated) (7 mice/group). Mice were sacrificed 7 weeks later. **A.** Brains (ventral view) were analyzed by means of a Lightools imaging system to detect fluorescence. Five independent examples are shown for each group. **B.** Metastatic burden in the brain was quantified by real-time PCR, according to [52]. Each bar represents mean ± SE. *Significance of difference *versus* FM-MSCs is *p* < 0.05 (Student *t* Test).

In breast cancer patients, the incidence of brain metastases as the site of first recurrence is increasing in recent times, because therapeutic monoclonal antibodies like trastuzumab are hampered by the blood-brain barrier [45]. We asked whether the i.v. administration of infected MSCs could exert therapeutic effects in a mouse model of brain metastasis induced by HER2^+^ MDA-MB-453-EGFP human breast cancer cells in NSG mice (Figure 4 and Figure 5). Tumor-free and metastasis-bearing mice received a single i.v. injection of R-LM249-infected FM-MSCs. First, we studied the systemic diffusion of cellular and viral genomes in NSG mice. The viral genome copy number in brains and in blood (Figure 4A-4B) was overall higher in NSG than in athymic nude, as expected given the stronger immune deficiency of NSG mice. The distribution to organs did not greatly differ between metastasis-bearing and tumor-free mice (Figure 4A-4B), and was very similar to that in athymic nude mice, except for blood and brains. Intravenous treatment with R-LM249-infected FM-MSCs inhibited the development of brain metastases. At necropsy, all mice treated with uninfected MSCs had prominent metastatic deposits in the brain (representative examples are shown in Figure 5A), whereas 40% of mice treated with R-LM249-infected MSCs had no or marginal brain involvement. To quantify the metastatic involvement in the brain, we used a human-specific, highly sensitive q-PCR assay, which showed that R-LM249, delivered *via* carrier cells, reduced by more than one-half the metastatic burden in the mouse brain (Figure 5B).

## DISCUSSION

The administration of o-HSVs by aid of carrier cells has been reported in a very limited number of preclinical studies, and only for the attenuated viruses now in clinical trials, with the aim to deliver them locally to glioblastomas, or to intraperitoneally diffuse tumors [38, 39]. It was not attempted for the systemic delivery. To our knowledge, the administration by way of MSCs has not been attempted with any of the fully retargeted/detargeted o-viruses.

Our aim was to set up an i.v. systemic delivery of the fully HER2-retargeted R-LM249 by means of carrier cells. We explored the feasibility of MSCs. An obstacle was that MSCs express negligible amounts of the cancer-specific HER2 receptor. We report that MSCs could be forcedly infected with the fully retargeted/detargeted o-HSV R-LM249 [21, 22], and provide proof-of-principle evidence of the efficacy of MSCs in delivering the retargeted o-HSV to metastatic sites, and in exerting therapeutic efficacy.

The forced infection of MSCs with R-LM249 was achieved by means of a short exposure of the virus-cell mixture to the fusogenic agent PEG6000, which increased the extent of infection from 4-8% to 34-38%, and, occasionally, to higher levels. This treatment greatly enhanced infection of all types of the MSCs tested, including MSCs from adipose tissue, dental pulp, bone marrow, etc. Thus, it appears to be of wide utility. The productive infection of MSCs resulted in some clear advantages. The payload of the virus that could be delivered to tumors was increased a few logs, relative to the input virus. Furthermore, the R-LM249-infected MSCs did not express the viral chimeric gD at their surface until 12 h after infection, and, thereafter, the expression was very low. This left a window of opportunity for the intracellular replicating virions, which were shielded from the attack to which extracellular virions are subject in the blood and parenchymal organs, as well as from the adaptive immune response.

Mice received a single i.v. administration of the infected MSCs. Taking into consideration that we administered 1×10^6^ cells, and the infected cell population represented about 30-40% of the total, this corresponds to a low amount of input virus. Clearly, the therapeutic effects reflected the advantages of the system, i.e., the cargo was multiplied, and was shielded from defensive systems. The therapeutic effect was observed in two murine model systems of metastatic dissemination. In the athymic nude mice the metastatic burden was at lungs, the organs where the viral and cellular genomes accumulated at higher amounts for anatomical reasons. In the NSG mice, a prominent site of metastatic accumulation was the brain. In the latter case, the effect occurred despite the fact that the viral and cellular genome copy numbers were significantly lower than in the lungs. The latter protection, along with the observation that viral genomes were maintained in the bloodstream, and organs, provides evidence that MSCs exert their carrier function beyond the lungs, which represent the first anatomical station. An issue regards the amount of infected MSCs that should be administered to humans. Here, infected MSCs were administered at a single dose of 3-4 × 10^5^ cells/mouse. Taking into account that the approximate factor for mouse to human converting doses is 12 [46], this would translate to a human equivalent of 7-9 × 10^7^ infected MSCs. This dose is lower than the clinical dose of 3 × 10^8^ MSCs envisioned in the translational study on fat tissue-derived MSCs, as carriers of o-measles virus [47]. Such amounts of cells, administered through a slow infusion, are unlikely to cause a high intravasal, or high local concentrations of MSCs, and therefore are unlikely to raise safety concerns. Indeed, similar amounts of MSCs were administered to patients with ischemic stroke, or multiple sclerosis and amyotrophic lateral sclerosis [48]. These amounts of cells do not raise feasibility concerns, since it has been estimated that the amount of cells that can be banked per patient is around 10^9^ [47].

We could not find any side effect of MSC administration in mice, and we found evidence of viral replication exclusively in neoplastic deposits. It should be kept in mind that, in the models system used here, only tumor cells expressed human HER-2, and that the antibody moiety used to redirect the virus does not cross-react with mouse HER-2. Our *in vitro* comparisons of normal and neoplastic cells lacking HER-2 amplification with neoplastic cells expressing very high levels of HER-2 as a consequence of gene amplification suggest that only the latter are significantly affected by viral replication [21]. In a translational perspective, we will have to study viral spread and replication in transgenic mice expressing human HER-2 [49], to evaluate the effect on normal tissues expressing low-level HER-2. Ultimately, only clinical studies will reveal what might happen in human organs that express low levels of HER-2, much as it happened with trastuzumab and other anti-HER-2 therapeutic antibodies. In particular cytotoxic conjugates, such as trastuzumab emtansine, demonstrated acceptable toxicities in clinical use [50].

It has been debated that a disadvantage of MSCs as carrier cells is that they can exert an enhancing effect on cancer cell replication [32, 51]. In our system we did not observe any significant increase in the number of metastases in mice treated with uninfected MSCs, as compared to untreated mice. Efforts are ongoing to improve the protocols for derivation of MSCs which exhibit a lower potential to promote tumors, hence the ultimate choice on which cells can be employed as carriers is still open [51].

Altogether, current data show that delivery of a retargeted o-HSV *via* MSCs is feasible and effective, and suggest that retargeted o-HSV may add to the list of oncolytic viruses under consideration for carrier-based systemic delivery.

## MATERIALS AND METHODS

### Isolation of MSCs

Fetal membrane (FM)-derived MSCs were isolated from term placentas, obtained from healthy donor mothers upon caesarean sections, with written informed consent and according to the policy of the Local Ethical Committee (Policlinico S. Orsola-Malpighi, protocol #1645/2014, reference 35/2014/U/Tess). Fetal membranes were separated from chorionic plate, and employed to isolate MSCs as described [41]. Dental pulp (DP) MSCs, bone marrow (BM) MSCs, and adipose tissues (Adipo) MSCs were isolated as described [42, 43], and cultured in Dulbecco's modified minimum essential medium (DMEM) containing 10% foetal bovine serum (FBS) (Invitrogen). Passages 4 to 9 MSCs were used throughout the experiments.

### Cells and viruses

Human ovarian cancer cell line SK-OV-3 [22] and human breast cancer cell lines MDA-MB-453-EGFP [52] exhibit very high HER-2 expression, and were cultured in RPMI (Roswell Park Memorial Institute) +10% FBS or DMEM plus 5% FBS, respectively [22]. J cells do not express HER-2 and gD receptors [44], and were cultured in DMEM supplemented with 5% FBS. The recombinant virus R-LM249, retargeted to HER2 [21] was grown and titrated in SK-OV-3 cells.

### Infection and virus replication

Cells were infected with R-LM249, at the indicated MOI, based on titers obtained in SK-OV-3 cells. All experiments were carried out with partially purified extracellular virions. For PEG6000-enhanced infection, virions were absorbed to MSCs at 37°C for 90 min; inoculum was removed; the virion-cell mixture was exposed for 20 sec to the fusogenic agent PEG6000 (Sigma-Aldrich) diluted at 40% in DMEM at 37°C, followed by extensive washes with serial dilutions of PEG6000 in PBS [53]. The extent of infection was monitored through detection of EGFP engineered in the viral genome, by flow cytometry using a BD ACCURI C6 (BD Becton Dickinson), or by fluorescence microscopy through an inverted microscope (Nikon TS100, Nikon Instruments). For determination of virus yield, SK-OV-3 and FM-MSCs cells in 12-well plates were infected with R-LM249 plus or minus PEG6000 treatment. Progeny virus (intracellular plus extracellular) was harvested at 24 h after infection and titrated in SK-OV-3 cells.

### R-LM249 spread from FM-MSCs to SK-OV-3 and MDA-MB-453 cells

FM-MSCs donor cells were infected with R-LM249 at 10 PFU/cell with PEG6000 treatment, followed or not by pH 3 wash. After viral adsorption, cells were incubated at 37°C for 4 h, trypsinized, resuspended in culture medium, serially diluted and seeded onto monolayers of acceptor SK-OV-3 cells or MDA-MB-453 cells in 12-well plates. After overnight incubation, cells were overlaid with 1% SeaPlaque Agarose (Lonza) in RPMI medium; plaques were scored by fluorescence microscopy at 24 h post infection.

### Flow cytometry analysis

HER2 expression in MSCs, SK-OV-3 and J cells was monitored by MGR2 monoclonal antibody (MAb) directed to human HER-2 (Enzo Life Sciences), F(ab')2-Goat anti-Mouse IgG (H+L) secondary Antibody, Alexa Fluor® 488 conjugate (Life Technologies). The cell surface expression of gD in R-LM249-infected FM-MSCs and SK-OV-3 cells was detected by means of MAb H170 [54] directed to linker between V_H_ and V_L__of the anti-HER2 single chain antibody, followed by PE (phycoerythrin-coupled) anti-mouse antibody.

### Mice

Athymic Crl:CD1-Foxn1^nu^ (referred to as nude) mice and NOD.Cg-Prkdc^scid^/II2rg^tm1Wjl^/SzJ NOD SCID gamma (referred to as NSG) mice were purchased from Charles River, and maintained under sterile conditions.

### Subcutaneous tumor model

Subcutaneous tumors in female nude mice were developed by injection of 2×10^6^ SK-OV-3 cells [21]. Five weeks later, FM-MSCs were infected ex vivo with R-LM249 as described above. Seven h later, infected cells, indicated as R-LM249 FM-MSCs, were trypsinized, washed, resuspended in PBS and injected s.c. Mice were sacrificed 144 h after receiving 10^6^ infected FM-MSCs. Resected tumors were cut in sections and examined using a Lightools imaging system (Lightools Research, Encinitas, CA) and photographed.

### Lung and brain metastases

For ethical reasons the experimental protocols excluded the possibility to analyze the overall survival of mice bearing metastases. Therefore, in all metastasis studies the mice were sacrificed after a predetermined time span, before metastases could cause distress or pain. For the development of lung metastases 2×10^6^ SK-OV-3 cells were injected i.v. in nude mice. A week later mice were treated with 10^6^ R-LM249-infected FM-MSCs, uninfected FM-MSCs or PBS, administered i.v. Mice were sacrificed about 10 weeks later and lung metastases were scored as described [55].

For the induction of brain metastases, MDA-MB-453-EGFP cells were injected i.v. (2×10^6^ cells) in female NSG mice. R-LM249-infected FM-MSCs were administered once, at 1×10^6^ cells in 0.4 ml PBS, six days after the injection of tumor cells. Control mice received an i.v. injection of uninfected FM-MSCs or PBS. Mice were sacrificed 7 weeks after tumor cell injection. Brains were analyzed under a Lightools imaging system and photographed. Metastatic burden was quantified by human centromeric DNA qPCR of brain genomic DNA, as described [52].

### Tissue distribution of R-LM249-infected FM-MSCs

Nude or NSG mice were inoculated with SK-OV-3 cells or MDA-MB-453-EGFP cells to develop metastases, as detailed above. After evaluating the presence of metastatic tumors in test animals, at 9 or 7 weeks after cancer cell inoculum for nude mice and for NSG mice, respectively, tumor-free and metastasis-bearing mice received an injection of 1×10^6^ R-LM249-infected FM-MSCs. Mice were sacrificed at indicated times after injection. Organs and blood were collected and immediately frozen or processed. DNA was extracted with NucleoSpin Tissue (Macherey-Nagel) accordingly to the manufacturer's instruction and subjected to qPCR. Determination of HSV genome copy number was as previously described [22]. The same samples were subjected to qPCR with primers For_Chr21 ATGCTGATGTCTGGGTAGGGTG and Rev_Chr21 TGAGTCAGGAGCCAGCGTATG, which amplify a 141-bp fragment [56]; the standard curve contained known quantities of human genomes (from 25000 to 1.6 human genome copies). Human genome copy number in brain tumors was described [22].

### Ethics statement

Isolation of MSC was performed according to the policy of the Local Ethical Committee (Ospedale Policlinico S. Orsola-Malpighi, protocol #1645/2014, reference 35/2014/U/Tess). All animal experiments were performed according to European directive 2010/63/UE, Italian laws 116/92 and 26/2014. Experimental protocols were reviewed and approved by the Institutional Animal Care and Use Committee (‘‘Comitato Etico Scientifico per la Sperimentazione Animale’’) of the University of Bologna, and forwarded to the Italian Ministry of Health with letters 19413-X/ 10 (Responsible Researchers G. Campadelli-Fiume and P. Nanni).

### Statistical analysis

Statistical analyses and determination of significance are reported in figure legends, as it applies.

## SUPPLEMENTARY MATERIAL FIGURES


