# Supplementary material for: Systemic delivery of HER2-retargeted oncolytic-HSV by mesenchymal stromal cells protects from lung and brain metastases
Source: Oncotarget. 2015 Sep 27;6(33):34774–87. doi: 10.18632/oncotarget.5793 (PMC4741489; doi:10.18632/oncotarget.5793)
Supplement: Supplementary file 1 [file oncotarget-06-34774-s001.pdf]

Systemic delivery of HER2-retargeted oncolytic-HSV by mesenchymal stromal cells protects from lung and brain metastases

Supplementary Material

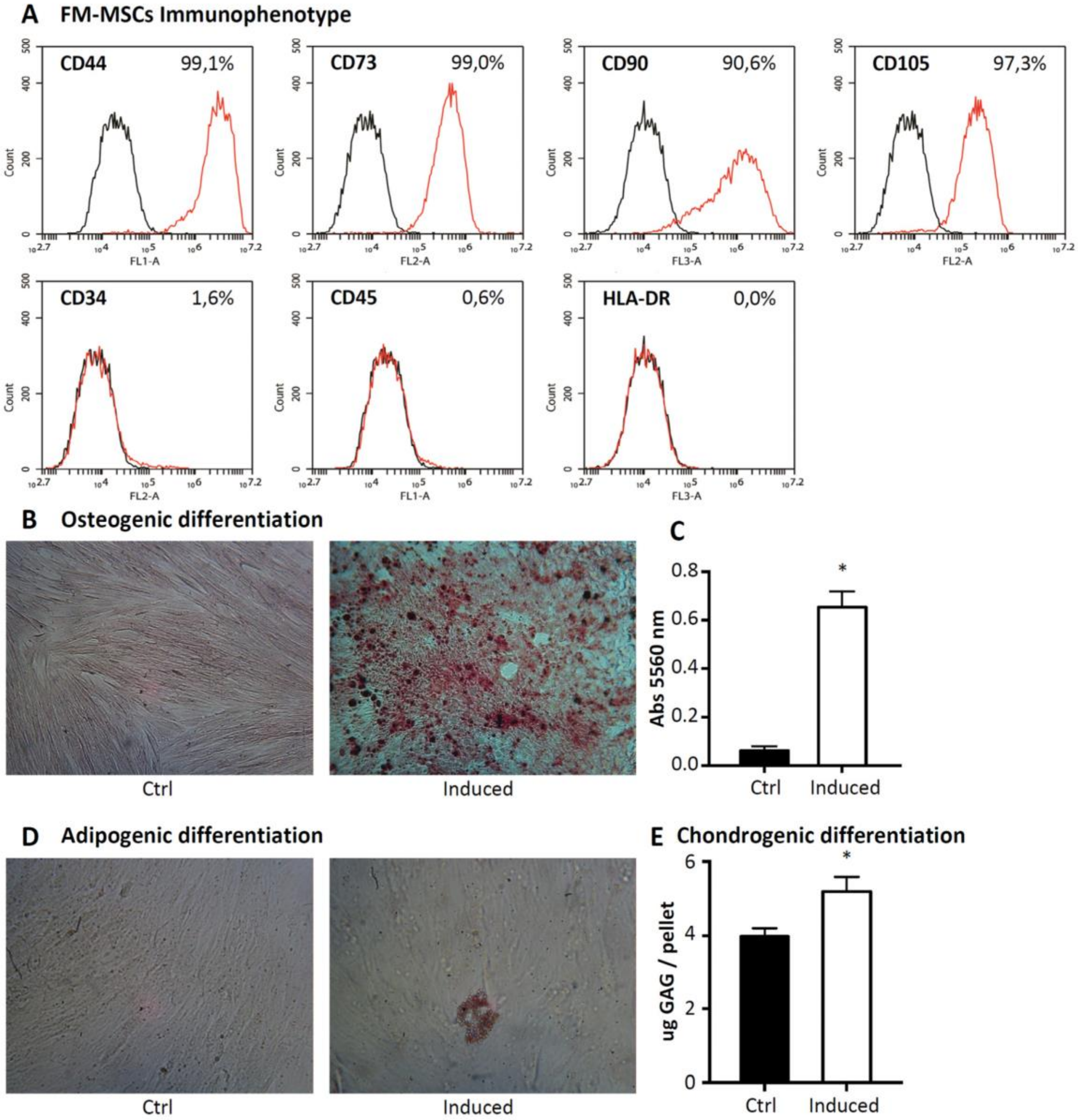

Figure S1: Characterization and *in vitro* differentiation of FM-MSCs. A. FM-MSCs, batch MF3619, express CD90, CD105, CD44, CD73 stromal markers, do not express the hematopoietic CD34 and CD45 markers, or HLAII DR marker, as assessed by flow cytometry by means of anti-CD34-Fluorescein isothiocyanate (FITC), anti-CD45-FITC, anti-CD44-FITC, anti-CD73-Phycoerythrin (PE), anti-CD90-phycoerythrin-cyanine 5 (PC-5), anti-CD105-PE, anti-HLA-DR-PE (all from Beckman Coulter or BD Becton Dickinson). Cells at passage 6th were tested for their ability to differentiate into the three classical lineages: osteogenic, adipogenic and chondrogenic.

Control cells were cultured in standard medium. **B., C.** Osteogenic differentiation was assessed using Alizarin Red staining (AR-S) and quantified by reading absorbance. **D.** Adipogenic differentiation was evaluated by Oil red O staining. **E.** Chondrogenic differentiation was determined by measure of glycosaminoglycan content measure. All analyses were carried out in triplicates. Histograms represent the mean of triplicates ± S.D. . Significance of difference was determined by t test.

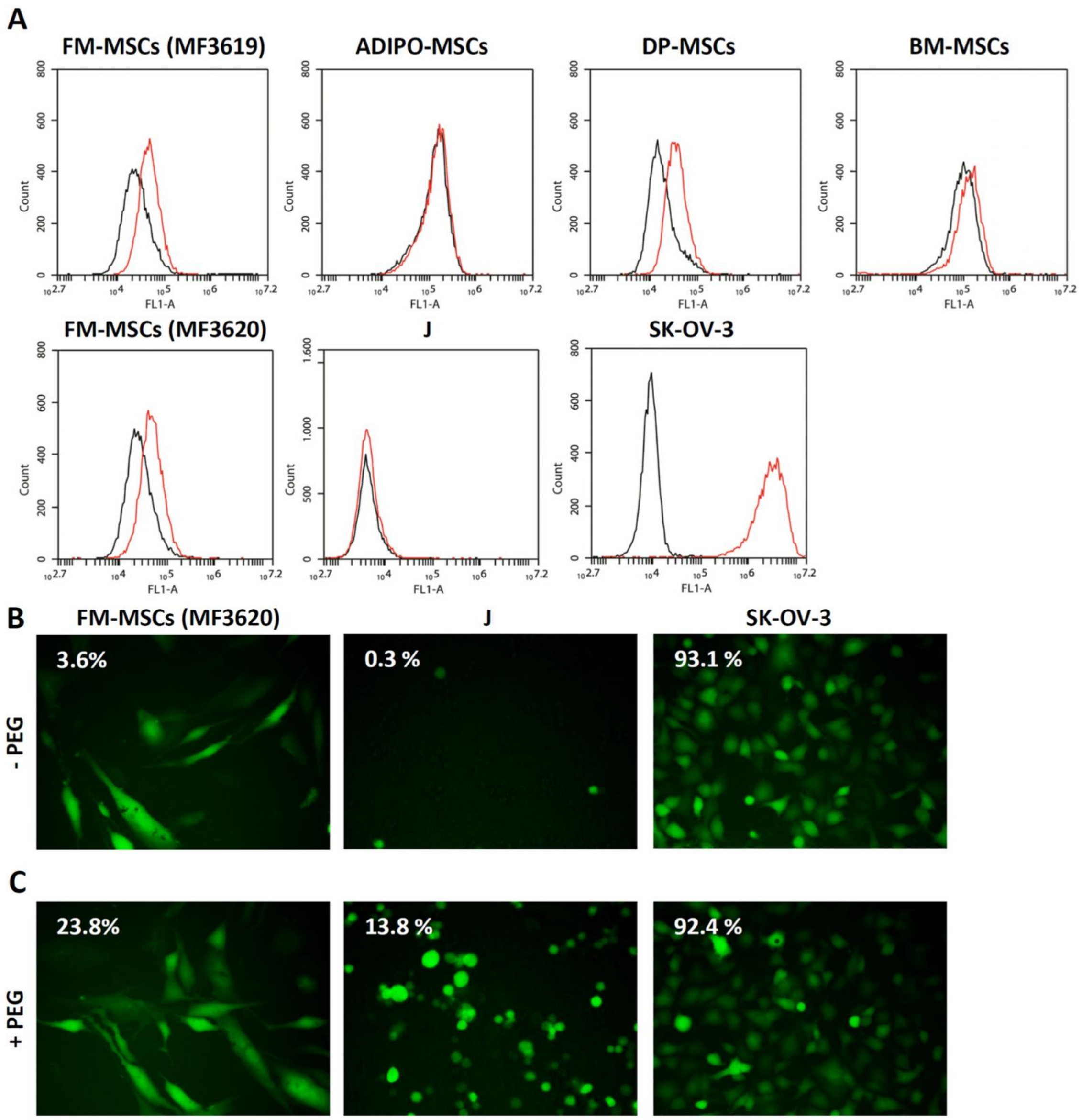

Figure S2: PEG6000-mediated enhancement of infection with R-LM249 of FM-MSCs and J cells. A. Cell surface expression of HER2 in FM-MSCs (batch MF3619 and MF3620), Adipo-MSCs, DP-MSCs, BM-MSCs, J cells and SK-OV-3 cells determined by flow cytometry by means of MAb MGR2. **B., C.** Enhancement of infection with R-LM249 in FM-MSCs by aid of PEG6000. Virions were absorbed to FM-MSCs, J, and SK-OV-3 at 10 PFU/cell. The virion-cell mixture was exposed for 20 sec to PEG6000. Infection was monitored through detection of EGFP engineered in the viral genome, by fluorescence microscopy and by flow cytometry. The percentage of infected cells is indicated as in Figure 1.

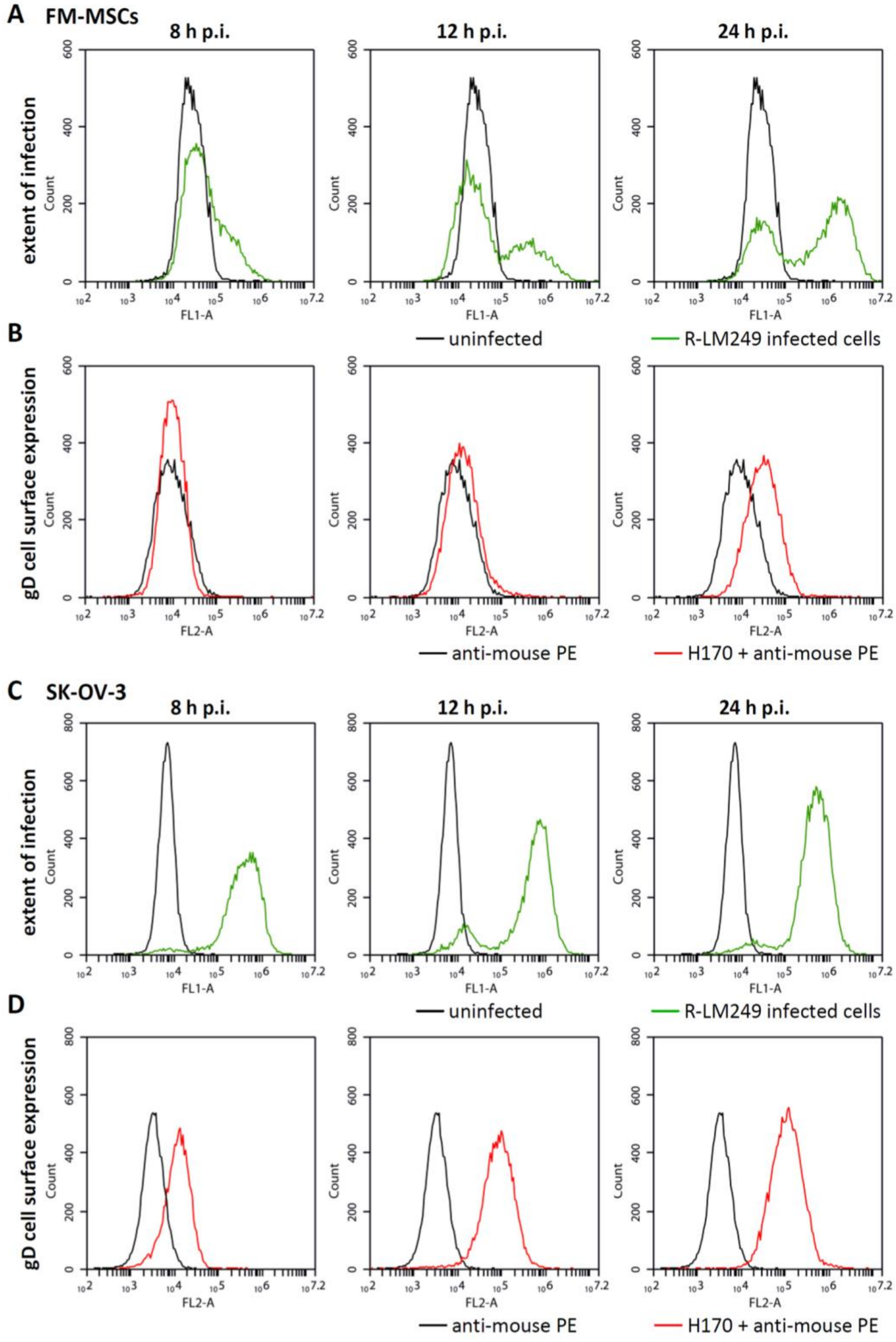

Figure S3: gD cell surface expression and extent of infection in FM-MSCs. A-D. FM-MSC and SK-OV-3 cells were infected with R-LM249, or uninfected, and harvested at 8, 12, 24 h after infection (p.i.). **A., C.** Extent of infection was quantified from the GFP marker encoded in the virus genome (green line), relative to uninfected cells (black line). **B., D.** Cell surface expression of the

chimeric scFv-gD was quantified in unfixed cells by means of the H170 MAb directed to the linker within the scFv (red line), relative to cells incubated with II antibody (black line).
